# Field Control Effect of *Telenomus remus* Nixon and *Trichogramma chilonis* Ishii Compound Parasitoid Balls against *Spodoptera frugiperda* (J. E. Smith)

**DOI:** 10.3390/insects15010028

**Published:** 2024-01-02

**Authors:** Xi Yuan, Yi Guo, Dunsong Li

**Affiliations:** Guangdong Provincial Key Laboratory of High Technology for Plant Protection, Institute of Plant Protection, Guangdong Academy of Agricultural Sciences, Guangzhou 510642, China; 13427690102@163.com (X.Y.); guoyi20081120@163.com (Y.G.)

**Keywords:** fall armyworm, egg parasitoids, biological control, combination ratio, field release

## Abstract

**Simple Summary:**

*Spodoptera frugiperda* is a devastating agricultural pest. *Telenomus remus* is an efficient egg parasitoid wasp for the control of *S. frugiperda*, but it has a limited host range and is expensive in application. In this manuscript, we combined *Trichogramma chilonis*, an easily available and cheap egg parasitoid wasp, with *Te. remus* in different ratios to form compound parasitoid balls and released them into a maize field to investigate their control efficacy against *S. frugiperda*. We found that compound parasitoid balls were more effective in the control of *S. frugiperda* than the release of only a single egg parasitoid wasp (*Te. remus* or *Tr. chilonis*). Moreover, the control effect against *S. frugiperda* could still retain more than 50% in the treatment, with 80% *Te. remus* and 20% *Tr. chilonis* and that with 20% *Te. remus* and 80% *Tr. chilonis* after 15 days of the release and given the higher cost of production of *Te. remus*, we concluded that the combination of 20% *Te. remus* and 80% *Tr. chilonis* was a more cost-efficient ratio for the control of *S. frugiperda*.

**Abstract:**

Although the release of egg parasitoids has proven to be an effective strategy for the control of the fall armyworm (FAW), a single egg parasitoid, however, has exposed some deficiencies in practice, and it is worthwhile to explore whether the combination of multiple parasitoid species released can be used to control FAW by adopting an inter- or intra-specific relationship. In this study, we released compound parasitoid balls of *Te. remus* and *Tr. chilonis* in maize fields to explore the effects of combinations in different proportions of these two egg parasitoids on the control of the FAW. The results showed that the release of compound parasitoid balls improved the control effect on the FAW compared to the release of only *Te. Remus* (100%) and only *Tr. Chilonis* (100%). The treatments released with compound parasitoid balls significantly increased the egg parasitism rate against the FAW, reduced its populations, and alleviated its damage to maize as compared to the treatment with only *Tr. chilonis* (100%) released, whereas there was no significant difference in the egg parasitism rate and FAW populations between the treatments with the release of only *Te. remus* (100%) and that of compound parasitoid balls. Among the compound parasitoid balls with different proportions of egg parasitoids, the treatment with 80% *Te. remus* and 20% *Tr. chilonis* and that with 20% *Te. remus* and 80% *Tr. chilonis* could still retain more than 50% of the control effect against the FAW after 15 days of release and given the higher cost of production of *Te. Remus*, our results suggested that the combination of 20% *Te. Remus* and 80% *Tr. Chilonis* is a more cost-efficient ratio for the control of the FAW. Our findings may provide a new perspective for the sustainable control of the FAW.

## 1. Introduction

The fall armyworm (FAW), *Spodoptera frugiperda* (Lepidoptera, Noctuidae), is one of the most destructive pests in agricultural production [1] and is extremely difficult to eradicate radically during production due to its high migratory ability [2], broad feeding habits [3], and the significant resistance it exhibits to a wide range of insecticides [4,5,6]. In addition, the larvae of the FAW are usually hidden in the inner leaves of the maize wheel, and the leaves are commonly covered with feces, which reduces the direct contact of the insects with the insecticide and, thereby, its efficacy [7]. Therefore, it is crucial to establish sustainable management strategies for this pest.

Biological control has proven to be an effective strategy for pest control due to its high environmental safety and effectiveness [8,9]. For example, biological control with the use of egg parasitoids has been developed for the management and control of the FAW [10,11]. Among the many egg parasitoids, *Trichogramma* spp. and *Telenomus remus* Nixon have attracted extensive attention in terms of their research and use [12,13,14]. It has been reported that *Trichogramma* spp. could parasitize the eggs of more than 200 insect species [15,16], and some of these egg parasitoids have been widely used for pest control in agricultural production around the world [17,18,19]. For example, the commercial release of *Trichogramma dendrolimi* Matsumura, *Trichogramma chilonis* Ishii, and *Trichogramma ostriniae* Pang et Chen has been successfully carried out on nearly 4 million hectares of maize fields per year in China [20]. The results of a maize field survey in India showed that *Tr. chilonis* egg parasitism rates on FAW were 15.81–23.87% [21], and field surveys in Tanzania indicated that *Trichogramma mwanzai* Schulten & Feijen have a high egg parasitism rate (70%) on FAW, which demonstrates promising control potential [22]. However, although some positive results have been observed in the biological control of the FAW by *Trichogramma* spp., the efficiency of its egg parasitism is also disturbed by several biotic and abiotic factors. Particularly noteworthy is the fact that the FAW usually lays eggs in multiple layers, with scales around and/or over the eggs when laid, which play an important role in protecting the eggs by providing a morphological or physical defense [10,23]. The presence of these scales alters the egg-laying behavior of the parasitoid wasps, thus affecting the control’s effectiveness [24,25]. In general, *Trichogramma* spp. can only parasitize eggs that are exposed in the outer layer and has difficulty in parasitizing eggs covered by heavy scales, as well as those in the inner layer of the egg mass [10]. For instance, the parasitism rates of *Trichogramma atopovirilia* Oatman & Platner on one-, two- and three-layered egg masses of the FAW were 66.2%, 45.2%, and 40.1%, respectively [26]. Therefore, in spite of *Trichogramma* spp. having good performance in terms of egg parasitism on the FAW, the weaknesses of *Trichogramma* spp. exposed to the egg defense (the existence of scales) of the FAW should be taken into account, and a new solution should be developed according to the control efficiency in the field.

*Te. remus*, a solitary parasitoid [27], due to its ability of strong parasitism [28], field dispersal [29], host searching [30], and, importantly, exhibiting high egg parasitism rates on superposed layers of the FAW [31], has become the most reported species in studies on FAW management and control [8]. The adult female of *Te. remus* can parasitize up to 220 eggs of the FAW in its lifetime [28], a single female of *Te. remus* for every five FAW eggs is sufficient to attain about 99% parasitism in maize fields [30], and its excellent control capability against the FAW has been verified in several Latin American countries [31,32,33]. However, global efforts to control the FAW through the release of *Te. remus* are still limited [14] due to their difficulty and high cost in large-scale rearing [31,34]. In addition, although the release of *Te. remus* is effective on *Spodoptera* spp. eggs, the parasitism rate on other important pests is not high [35]. However, a pest outbreak is not often the result of a single species, and farmers are often faced with multiple infestations occurring at the same time. Based on this, some studies have attempted to combine two egg parasitoids, concentrating their main advantages in the same biological control product to overcome the disadvantages exposed by a single egg parasitoid. A laboratory study indicated that the combination of *Trichogramma pretiosum* Riley (90%) and *Te. remus* (10%) had the same parasitism potential on FAW egg masses compared to *Te. remus* only (100%) [36], which provides a new perspective on balancing the cost and efficiency of pest control, i.e., the same effect can be achieved by using more of the cheap and easy-to-produce egg parasitoids supplemented with little expensive parasitoid wasps. However, there are still fewer reports on the control efficacy of egg parasitoids through the combinations of multiple parasitic wasps in the field against the FAW, and the optimal combination ratio, the release of methods, time and frequency, and number of release sites among egg parasitoid species need to be explored urgently [31].

As a species of *Trichogramma* spp., the gregarious parasitoid *Tr. Chilonis* [37] not only shows potential in the control of the FAW, but also shares the disadvantages of *Trichogramma* spp. mentioned above. In this study, we released parasitoid balls of a combination of *Te. remus* and *Tr. chilonis* in maize fields to investigate the control effectiveness of different combination proportions of egg parasitoids against the FAW and select the optimal combination ratio. Given the biological characteristics of the two egg parasitoid wasps, we hypothesized that the release of compound parasitoid wasps would be more effective than a single parasitoid wasp in the field and that the control efficacy of the combination of 80% *Te. remus* and 20% *Tr. chilonis* was better than the others. Our findings may provide a reference for the control of the FAW and the development of integrated pest management strategies.

## 2. Materials and Methods

### 2.1. Insect Sources and Rearing

**Figure 1:**
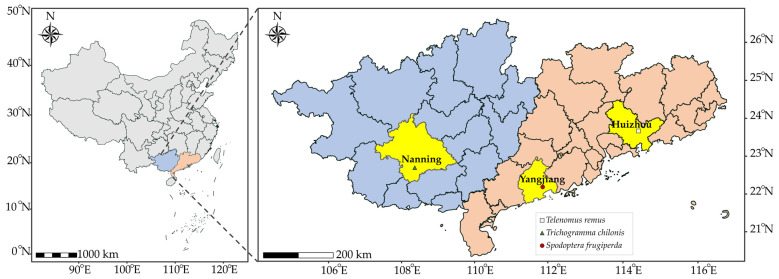
Location of the Guangxi Zhuang autonomous region and Guangdong Province in China and the specific location of the insect collection sites in the corresponding regions.

*Te. remus*, *Tr. Chilonis*, and the FAW were collected from different areas (Figure 1). *Te. remus* were collected from the parasitized egg masses of the FAW in maize fields in Lianghua Town (23°12′ N, 114°68′ E), Huizhou City, Guangdong Province, China, identified and purified indoors, and bred with *Spodoptera litura* (Fabricius) eggs for multiple generations for experiments. *Tr. chilonis* was derived from the parasitized egg masses of *Proceras venosatum* (Walker) and *Chilo infuscatellus* (Snellen) in sugarcane fields in Nanning City (22°61′ N, 108°24′ E), Guangxi Zhuang Autonomous Region, China. After being identified and purified in the laboratory, *Tr. chilonis* was propagated over several generations with *Corcyra cephalonica* (Stainton) eggs for subsequent experiments.

The FAW came from the green storage maize field in Yangjiang City (21°93′ N, 111°90′ E), Guangdong Province, China, and the maize variety was Guidan 0180. The FAW was raised for multiple generations in an artificial climate incubator (RDZ-300D-4W, Ningbo Jiangnan Instrument Factory, Ningbo, China) in the laboratory for experiments. The culture temperature was 27 ± 1 °C, the relative humidity was 85% ± 10%, the photoperiod L:D = 14:10, the light intensity was 8000 lx, and the feeding method of the FAW larvae was that of Yuan et al. [38].

### 2.2. Experimental Design

#### 2.2.1. Field Experiment

The experimental site was located in the organic maize cultivation base of Liumuyuan Organic Farm (23°16′ N, 114°71′ E), Lianghua Town, Huizhou City, Guangdong Province, China. The maize cultivar at the experimental site was Jinyinsui 2, with planting densities of 45,000–50,000 plants/ha, and the preceding crop was maize. At the time of releasing the parasitoid balls, the maize plants were at the big flare period, and the height of the plants was about 1 m. According to the different combination ratios of parasitoid balls, seven treatments were set up, with three replications for each treatment, and the treatment without releasing parasitoid balls as the field control, and a total of 21 plots were set up in this experiment (Table 1). Each plot covered an area of 100 m^2^ (10 m × 10 m), and the plots were arranged in a completely randomized design. The distance between plots was more than 100 m, and all plots had the same varieties, fertilization, irrigation, and other field management practices. The experiment was repeated three times at the Liumuyuan Organic Farm in March, July, and October 2023, with the weather during the experiment period being sunny to cloudy weather with occasional showers, rainfall of 15 mm or less, air temperature of 22~34 °C, and relative humidity of 58~83%.

Before the trial began, a strict management measure was implemented to control pests in the experimental field. Insecticides were applied every two days during the vegetative growth period of maize after sowing. The insecticide was a combination of 5% emamectin benzoate microemulsion produced by Qingdao Runsheng Agrochemical Co., Ltd. (Qingdao, China), and 200 g/L chlorantraniliprole suspension produced by FMC Corporation, USA. When spraying insecticides, it is necessary to uniformly spray the front and back of maize leaves, leaf sheaths, and stalks and then observe the field daily to check whether there are FAWs and their natural enemies. If they were found, the insecticide was sprayed on the same day to ensure that there were no FAWs and their natural enemies in the experimental field, and the damage rate of the maize plants before the release of parasitoid balls was controlled to be “0”.

**Figure 2:**
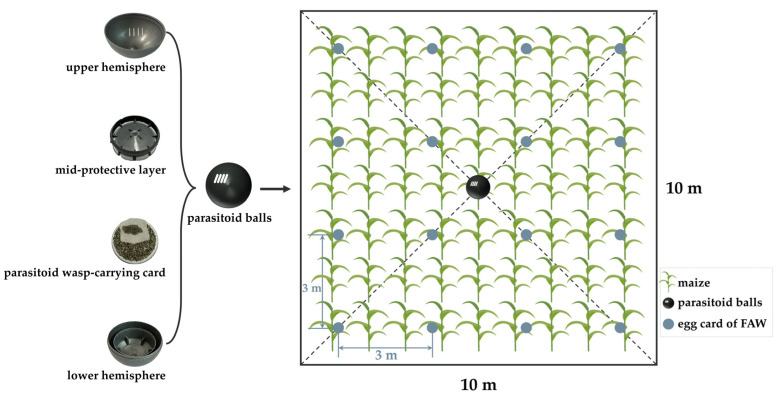
The main structures of parasitoid balls and the field layout of parasitoid balls and *Spodoptera frugiperda* egg cards.

The compound parasitoid balls were composed of four parts, namely, the upper hemisphere, the mid-protective layer, the lower hemisphere, and the parasitoid wasp-carrying card (Figure 2). To make the parasitoid balls by hand, the lower hemisphere was opened upwards and filled with *Tr. chilonis*, then filled with *Te. remus*, covered with the mid-protective layer, and finally capped with the upper hemisphere; we checked for sealing of the ball and completed the process of the compound parasitoid balls. A single parasitoid ball was released at the center of the plot (diagonal intersection), and the parasitoid ball was placed at the trumpet of the heart leaf of the maize plant, about 0.8 m above the ground, and one egg mass was hung at intervals of 3 m in each plot (10 m × 10 m), with a total of 10 egg masses in each plot. Egg masses were suspended from the abaxial leaf veins of the maize leaves and fixed with staples to avoid falling, the egg cards were about 1 m from the ground, and the plants at the point where the egg cards were released were marked clearly to facilitate retrieval after 5 days.

#### 2.2.2. Field Survey

On the 5th day after release, all of the egg cards were collected and brought back to the laboratory, and each egg mass was marked and numbered, then packed into 50 mL plastic tubes individually sealed with breathable paper, and placed in the artificial climate incubator at a temperature of 27 ± 1 °C, a relative humidity of 85 ± 10%, a light intensity of 8000 lx, and a photoperiod of 14L:10D. After the egg mass developed and the adult parasitoid emergence, the parasitism of the egg mass was investigated.

On the 5th, 10th, and 15th day after release, the maize plants were investigated for the damage index caused by the FAWs and the number of the FAW population. Five rows of maize plants were randomly selected in each plot and 10 plants were surveyed consecutively in each row. A total of 50 maize plants were investigated in each plot, and the data of the plant inverted 3-leaf damage level were recorded. The damage level was classified as 0–9, and the standard used referred to that of Liu et al. [39]. The control effect was also calculated according to the methods of Liu et al. [39]. Considering that the number of live insects before the release of parasitoid balls cannot be zero as the denominator for the calculation of the rate of population reduction, it was calculated as 1 insect per 100 plants in calculating the reduction rate of the insect population. The calculation formula is as follows:

(1) $$\mathrm{DI}=\frac{\sum {\mathrm{DL}}_{p}\times {\mathrm{DL}}_{l}}{9\times T_{pv}}$$

(2) $$\mathrm{CE}=\frac{{\mathrm{DI}}_{\mathrm{ck}}-{\mathrm{DI}}_{t}}{{\mathrm{DI}}_{\mathrm{ck}}}\times 100\%$$

(3) $$\mathrm{PCR}=\frac{{\mathrm{NI}}_{b}-{\mathrm{NI}}_{a}}{{\mathrm{NI}}_{b}}\times 100\%$$

(4) $$\mathrm{CPCR}=\frac{{\mathrm{PCR}}_{t}-{\mathrm{PCR}}_{\mathrm{ck}}}{{1-\mathrm{PCR}}_{\mathrm{ck}}}\times 100\%$$

where *DI* is the damage index; *DL_p_* is the plants for each damage level; *DL_l_* is the levels for each damage level; 9 is the defined nine damage levels; *T_pv_* is the total number of plants investigated; *CE* is the control effect; *DI_ck_* is the damage index of the blank control plots; *DI_t_* is the damage index of the treatment plots; *PCR* is the population reduction rate; *NI_b_* is the number of live insects before the release of parasitoid balls; *NI_a_* is the number of live insects after the release of parasitoid balls; *CPCR* is the corrected population reduction rate; *PCR_t_* is the population reduction rate in the treatment plots; *PCR_ck_* is the population reduction rate in the blank control plots.

### 2.3. Statistical Analysis

One-way analysis of variance (ANOVA) was performed using SPSS 25.0 (IBM, Armonk, NY, USA), and Duncan’s new complex polar difference method was used for multiple comparisons. For the variables presented as numbers with percentages, statistical significance was tested using the binomial test. All graphics were drawn in Microsoft Excel 2016 (Microsoft Corporation, Redmond, WA, USA).

## 3. Results

### 3.1. Emergence Rate of Compound Parasitoid Balls and the Egg Parasitism Rate on the FAW

**Figure 3:**
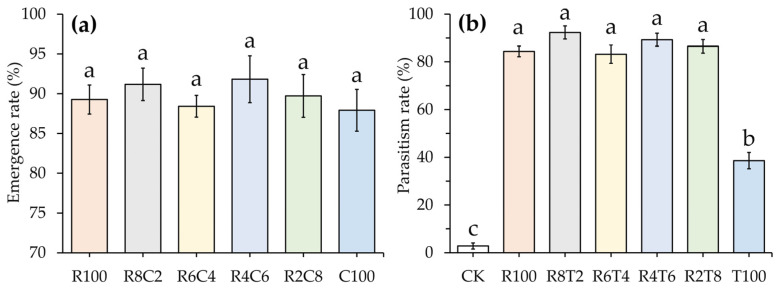
Emergence rate of compound parasitoid balls and their egg parasitism rate on the FAW in maize fields. (**a**) Emergence rate; (**b**) parasitism rate. Error bars indicate the mean ± standard error (SE%), and the different lowercase letters indicate significant differences at the *p* < 0.05 level as tested by Duncan’s new complex polarity test. R_100_, R_8_C_2_, R_6_C_4_, R_4_C_6_, R_2_C_8_, and C_100_ indicate the release of parasitoid balls with only *T.remus* (100%), 80% *Te. remus* combined with 20% *Tr. chilonis*, 60% *Te. remus* combined with 40% *Tr. chilonis*, 40% *Te. remus* combined with 60% *Tr. chilonis*, 20% *Te. remus* combined with 80% *Tr. chilonis*, and only *Tr. chilonis* (100%), respectively.

The emergence rate of compound parasitoid balls was not significantly different between the treatments in which parasitoid balls were released, with all emergence rates above 87.9% (Figure 3a). The treatment with only *Tr. chilonis* (100%) released was significantly lower in terms of the egg parasitism rate (38.58%) on the FAW than the other treatments with the release of parasitoid balls (*p* < 0.05, Figure 3b); however, the differences in the egg parasitism rates among the other treatments with parasitoid balls released were not significant, with the highest egg parasitism rate (92.3%) in the treatment of 80% *Te. remus* combined with 20% *Tr. chilonis*, and the egg parasitism rate in the treatments of parasitoid ball released was significantly higher than that in the treatment without parasitoid ball released (2.81%) (*p* < 0.05).

### 3.2. Variation in FAW Populations and Damage Index

**Figure 4:**
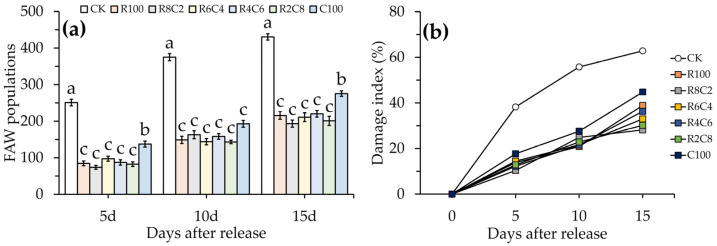
Variation in FAW populations and the damage index after the release of compound parasitoid balls. (**a**) FAW populations; (**b**) damage index. Error bars indicate the mean ± standard error (SE%), and the different lowercase letters in the same period indicate significant difference at the *p* < 0.05 level as tested by Duncan’s new complex polarity test. R_100_, R_8_C_2_, R_6_C_4_, R_4_C_6_, R_2_C_8_, and C_100_ indicate the release of parasitoid balls with only *T.remus* (100%), 80% *Te. remus* combined with 20% *Tr. chilonis*, 60% *Te. remus* combined with 40% *Tr. chilonis*, 40% *Te. remus* combined with 60% *Tr. chilonis*, 20% *Te. remus* combined with 80% *Tr. chilonis*, and only *Tr. chilonis* (100%), respectively.

After the FAW egg cards were released in the maize field, the number of FAWs in the field increased rapidly in a short period of time, from 251 insects 5 days after release to 430 insects 15 days later (Figure 4a). The release of parasitoid balls significantly limited the growth of FAW populations. Approximately 15 days after release, the average number of FAWs in all of the treatments with parasitoid balls released was 219 insects, which was significantly lower than the 430 insects in the treatment without any released (*F* = 13.749, df = 20, *p* = 0.021). Among the treatments of releasing parasitoid balls, the number of FAWs was considerably higher in the treatment with only *Tr. chilonis* (100%) released than in the other released treatments both after 5 days (*F* = 13.256, df = 17, *p* = 0.022), 10 days (*F* = 13.923, df =17, *p* = 0.024), and 15 days (*F* = 12.859, df = 17, *p* = 0.026) of release, and there was no significant difference among the other released treatments. The treatment of 80% *Te. remus* combined with 20% *Tr. chilonis* had a lower number of FAWs than the other released treatments 15 d after the release of the parasitoid balls.

The damage index is basically consistent with the changes in FAW populations, with a progressive increase in damage to maize caused by FAWs over time (Figure 4b). The treatments with the release of parasitoid balls alleviated the damage to the maize caused by FAWs. No matter whether it was 5 days (*F* = 0.462, df = 20, *p* = 0.023), 10 days (*F* = 0.623, df = 20, *p* = 0.016), or 15 days (*F* = 0.417, df = 20, *p* = 0.020) after the parasitoid balls were released, the damage index in the treatments with the release of parasitoid balls was significantly lower than that of the treatment with none released. There were no significant differences in the damage index between all of the released treatments after 5 days (*F* = 2.762, df = 20, *p* = 0.102) and 10 days (*F* = 2.374, df = 20, *p* = 0.118) of release; however, after 15 days of parasitoid balls release, the damage index of the treatment with only *Tr. chilonis* (100%) was significantly higher than that of the other released treatments (*F* = 0.276, df = 20, *p* = 0.045). The lowest damage index was observed in the treatment of 80% *Te. remus* coupled with 20% *Tr. chilonis.*

### 3.3. Corrected Population Reduction Rate and Control Effect

The corrected population reduction rate and control effect provide a more direct view of the management efficacy of releasing egg parasitoids on the FAW. Approximately 5 days after releasing the parasitoid balls, the corrected population reduction rate and control effect were 76.83% and 68.52% on average, respectively (Table 2). However, the control effect decreased gradually over time, dropping to 28.8–55.2% after 15 days. Meanwhile, the control effect among treatments showed more differences, with 80% *Te. remus* combined with 20% *Tr. chilonis* being significantly higher than that of releasing only *Te. remus* (100%) (*p* < 0.05) and releasing only *Te. remus* (100%) (*p* < 0.05).

## 4. Discussion

Although some important advances have been made in the use of egg parasitoids for the control of the FAW, the control effect of egg parasitoids on the FAW, however, is subject to a combination of internal and external factors, such as the species of egg parasitoids, the number of releases in the field, the release technology (spatial–temporal management), the characteristics of the pests, crop management (insecticide use) [13], etc. As the natural egg parasitoids of the FAW in the field, *Te. remus* and *Tr. chilonis* have received widespread attention in previous studies, and the two egg parasitoids have both advantages and disadvantages in the control of the FAW [13,21,40]. In this study, the two egg parasitoids were deployed in different ratios to form different combinations in order to explore whether the advantages of the two egg parasitoids could fully play a role in the control and management of the FAW while avoiding the disadvantages caused by the release of a single egg parasitoid.

In this study, we observed that the egg parasitism rate on the FAW for the release of only *Tr. chilonis* (100%) was only 38.58% and was significantly lower than that of 84.35% for the release of only *Te. remus* (100%), which may be closely related to the egg-laying characteristics of the FAW. The FAW usually lays its eggs in multiple layers and uses the scales from its body as a cover to protect the egg masses from natural enemies [41].Previous research has shown that the scales on the egg masses of the FAW are an important barrier to egg parasitism by some *Trichogramma* spp., which can only parasitize the eggs on the periphery of the egg masses and has difficulty in parasitizing the eggs below a large number of scales and those in the inner layers of the egg masses [26]. For example, *Tr. pretiosum* exhibited an egg parasitism rate of 19.90% on the FAW in the presence of scales, which was significantly lower than the rate of 46.00% without scales [10]. Nevertheless, *Trichogramma* spp. are still widely used in the biological control of pests due to their efficient parasitism on *Helicoverpa* spp. [42,43] and a broad range of host species [44]. In contrast, *Te. remus* is well adapted to the characteristics of FAW egg masses, which can crawl into the scale-covered egg layer and parasitize the eggs in the inner layer of the egg mass, with comparable parasitism rates on both scaled and non-scaled egg masses [10]. However, the natural host range of *Te. remus* is more limited than that of *Tr. chilonis*, and it may be difficult to release *Te. remus* alone to achieve the desired pest control when multiple pests occur in parallel, as it has been found that outbreaks of FAWs are often accompanied by other pests (e.g., *Helicoverpa* spp.) [13]. As a result, several studies have attempted to combine the advantages of two egg parasitoids into a single biocontrol product and use it for FAW control [36]. We found that the release of compound parasitoid balls significantly increased the egg parasitism rate on the FAW compared to the release of a single parasitoid wasp (*Te. remus* or *Tr. chilonis*), which is consistent with the observation of Xie et al. [45] in that the efficiency of parasitism by the two species of egg parasitoids together (89.5%) was higher than that by either species individually (77.4% for *Te. remus* and 73.6% for *Tr. chilonis*). Previous studies have argued that synergism between two organisms (predators) only occurs when one of the predators makes the prey more vulnerable to attack by the other predator [46]. However, another study found that some parasitoid wasps could recognize FAW eggs that had already been parasitized by others [47]. For example, *Te. remus* and *Tr. pretiosum* were able to recognize eggs parasitized by each other, and there was no *Te. remus* emergence when the FAW eggs were previously exposed to *Tr. pretiosum* [48], and this phenomenon might be explained by competition for host resources among parasitoid wasps. This phenomenon can be explained by competition for host resources among parasitoids since a single parasitoid often fails to occupy all of the egg particles of an egg mass, and this competition in turn promotes parasitism on the egg mass by different parasitoids, thus increasing the utilization rate of the egg mass.

The cost of production is an important factor affecting the large-scale application of biological control agents. In this study, we also found no significant differences between different combinations of proportions of parasitoid balls in terms of emergence rate, egg parasitism on the FAW, and the FAW populations. Even after 15 days of release, there were no significant differences in the control effect of the treatments with the release of compound parasitoid balls, except for the treatment with 40% *Te. remus* and 60% *Tr. chilonis*. Although the control effects achieved were generally close to each other, there was a large difference in the cost of production. Previous studies have indicated that the mass rearing of *Te. remus* is still limited due to the difficulty of its development in the eggs of its natural host and the heavy labor costs involved in its production [49]. The cost of *Te. remus* production is USD 0.0004 per wasp based on the eggs of the FAW, and the cost is USD 0.0002 per wasp when using the eggs of *C. cephalonica* [34]. The high cost of production has motivated many researchers to develop new host alternatives and production techniques [11,50]. In contrast, the production of *Trichogramma* spp. is much more mature, and in the early 1990s, an automated production line with an annual capacity of 40 billion *Trichogramma* was set up in Jilin Province, China [51]. In recent years, the production and application of *Trichogramma* has become more cost-effective, thanks to the improvements in *Trichogramma* production technology, which produces approximately 800 million to 1 billion wasps per day from biological stations [52]. Therefore, given the field control effect of compound bee balls and the production costs of these two egg parasitoids, we believe that 20% *Te. remus* and 80% *Tr. chilonis* has more promotion value in the practice of FAW control.

Although we have conducted some useful explorations into the release of compound parasitoid balls for the control of the FAW, there are still more problems that deserve to be explored. For example, we found that the field efficacy of releasing compound parasitoid balls against the FAW declined over time. Faced with the declining field efficacy, previous studies have addressed the problem through improved release technology [9,13,31], and the combination of chemical control [53]. However, these methods have rarely been reported in studies of the release of compound parasitoid balls for the control of agricultural pests, and more research is still required on the combinations between different egg parasitoid species and their release techniques. In conclusion, we investigated the field efficacy of the release of compound parasitoid balls for the control of the FAW, which provides a new perspective for the sustainable control of the FAW, and it is still worthwhile to input more efforts in the future for in-depth research.

## 5. Conclusions

Our results indicate that the release of compound parasitoid balls has a greater control effect against the FAW in maize fields compared to the release of a single egg parasitoid (*Te. remus or Tr. chilonis*). Compared with the release of only *Tr. chilonis* (100%), the release of compound parasitoid balls significantly increased the egg parasitism rate on the FAW, reduced FAW populations, and relieved the damage to maize caused by the FAW. After 15 days of the release of compound parasitoid balls, they were equally effective in the control of the FAW in the treatment with 20% *Te. remus* and 80% *Tr. chilonis* and that of 80% *Te. remus* and 20% *Tr. chilonis*, even though the treatment with 80% *Te. remus* and 20% *Tr. chilonis* was higher in the control effect; given the higher cost of production of *Te. remus*, we concluded that 20% *Te. remus* and 80% *Tr. chilonis* was a more cost-effective treatment for the control of the FAW. Our finding may provide valuable insight into cost control and the development of sustainable management strategies for the control of the FAW.

## Figures and Tables

**Table 1:** Proportion of *Te. remus* and *Tr. chilonis* in the compound parasitoid balls.

Treatment	No. of *Te. remus*	No. of *Tr. chilonis*	*Te. remus* and *Tr. chilonis* Ratios
CK	0	0	0:0
R_100_	1000	0	100%:0
R_8_C_2_	800	200	80%:20%
R_6_C_4_	600	400	60%:40%
R_4_C_6_	400	600	40%:60%
R_2_C_8_	200	800	20%:80%
C_100_	0	1000	0:100%

**Table 2:** Corrected population reduction rate and control effect after the release of parasitoid balls.

Treatment	Corrected Population Reduction Rate (%)			Control Effect (%)		
	5 d	10 d	15 d	5 d	10 d	15 d
R_100_	82.4	−17.46	−26.69	71.86 ± 2.37 a	62.62 ± 2.74 a	38.11 ± 2.49 c
R_8_C_2_	86.8	−47.95	−3.9	75.86 ± 2.83 a	55.44 ± 2.57 ab	55.20 ± 2.41 a
R_6_C_4_	75.2	1.72	−28.07	66.20 ± 2.47 b	61.29 ± 2.79 a	47.54 ± 2.83 ab
R_4_C_6_	78.2	−21.07	−21.43	68.02 ± 2.63 ab	61.48 ± 2.52 a	42.24 ± 2.47 c
R_2_C_8_	80.2	−16.26	−21.97	72.48 ± 2.31 a	59.05 ± 2.38 a	51.97 ± 2.06 a
C_100_	58.2	6.57	−24.91	53.71 ± 2.34 c	50.55 ± 2.74 b	28.80 ± 2.49 d

Note: Data in the table are the mean ± standard deviation. Different lowercase letters in the same column indicate significant differences at the *p* < 0.05 level as tested by Duncan’s new complex polarity test. R_100_, R_8_C_2_, R_6_C_4_, R_4_C_6_, R_2_C_8_, and C_100_ indicate the release of parasitoid balls with only *T. remus* (100%), 80% *Te. remus* combined with 20% *Tr. chilonis*, 60% *Te. remus* combined with 40% *Tr. chilonis*, 40% *Te. remus* combined with 60% *Tr. chilonis*, 20% *Te. remus* combined with 80% *Tr. chilonis*, and only *Tr. chilonis* (100%), respectively.

## Data Availability

The data presented in this study are available from the corresponding author upon request.
